# Impact of Traumatic Injuries to Primary Teeth on the Development of Permanent Dentition: Findings From a Retrospective Cohort

**DOI:** 10.1111/edt.70004

**Published:** 2025-07-25

**Authors:** Vanessa Polina Pereira da Costa, Fernanda Vieira Almeida, Giulia Tarquinio Demarco, Maria Giulia Larroque Silva da Motta, Caroline de Oliveira Langlois, Alexandre Emidio Ribeiro Silva, Marilia Leão Goettems

**Affiliations:** ^1^ Post‐Graduate Program in Dentistry, Department of Social and Preventive Dentistry Federal University of Pelotas Pelotas Brazil; ^2^ Post‐Graduate Program in Dentistry Federal University of Pelotas Pelotas Brazil; ^3^ Graduate in Dentistry Federal University of Pelotas Pelotas Brazil; ^4^ School of Dentistry Federal University of Pelotas Pelotas Brazil

**Keywords:** children, cohort study, primary dentition, tooth injuries

## Abstract

**Objective:**

This retrospective cohort study aimed to analyze the occurrence of sequelae in permanent teeth resulting from traumatic injuries to primary dentition among children treated over a 14‐year period at a specialized Dental Trauma Center.

**Methods:**

Data from 140 children with complete records on trauma and sequelae were evaluated. Poisson regression was applied to calculate relative risks (RR) with 95% confidence intervals (CI).

**Results:**

Out of the 244 primary teeth affected by trauma, 81 (33.2%) exhibited sequelae in their permanent successors. The majority of sequelae were mild, with enamel discoloration being the most common type. Intrusive luxation and avulsion were the main causes of sequelae. Factors influencing sequelae included age, trauma location, and trauma type. Children aged 2–4 years (RR = 0.46; 95% CI: 0.26–0.85) and those older than 4 years (RR = 0.30; 95% CI: 0.15–0.60) experienced less sequelae compared to children under 2 years of age (*p* = 0.003). Dental injuries sustained outside the home were linked to a higher incidence of sequelae (RR = 2.07; 95% CI: 1.16–3.66) compared to those occurring at home (*p* = 0.012). Additionally, supporting tissue injuries posed a significantly higher risk (RR = 2.79; 95% CI: 1.12–6.93) compared to hard tissue injuries (*p* = 0.027).

**Conclusions:**

Sequelae in permanent teeth were commonly observed after primary dentition trauma, with the majority being classified as mild. Trauma to supporting tissues, younger age (< 2 years), and injuries occurring outside the home were associated with the development of sequelae in permanent teeth.

## 1 Introduction

Over one billion individuals globally have experienced traumatic dental injuries (TDI), including approximately 180 million children, with 22.7% of children worldwide affected by dental trauma in primary dentition [1]. TDI in primary dentition, though less explored compared to permanent dentition, holds particular importance as such injuries can lead to sequelae in both the primary teeth and the developing permanent dentition [2], requiring long‐term follow‐up. Research indicates that the prevalence of developmental issues after traumatic dental injuries in primary teeth varies between 12% and 74% [3], largely influenced by the proximity of the apex of the primary tooth root to the underlying permanent tooth germs [4]. This wide‐ranging condition can vary from enamel hypocalcifications to an arrest of the permanent tooth development [5]. Trauma to primary teeth can directly impact the development of permanent successors, leading to hypoplasia, germ displacement, or a variety of structural and functional defects, ranging from mild to severe. In certain instances, the effects of oral and dental trauma become evident only upon the eruption of permanent incisors, leading to ectopic eruption, misalignments, and other developmental complications [6, 7, 8, 9].

Earlier studies have linked a child's age at the time of trauma to the likelihood of sequelae occurring in permanent teeth after dental trauma in primary dentition [7, 10, 11]. Variables like the extent of root resorption in the traumatized deciduous tooth and the developmental stage of the successor tooth may impact the emergence of sequelae [2, 6, 12]. Regarding the type of TDI, intrusion and avulsion injuries are most commonly associated with disturbances in the permanent successor [13]. Noteworthy, a previous study found that all types of TDI were associated with sequelae in permanent successors [14].

Treatment of sequelae may be time‐consuming, require a multidisciplinary approach, and involve high costs [13]. A study using thematic maps showed a higher dental trauma prevalence in areas of unfavorable socioeconomic conditions [15], suggesting that place of residence may influence trauma occurrence. Considering that variations in access to emergency dental care, socioeconomic status, and parental awareness might influence both the frequency and severity of dental trauma, as well as the follow‐up care, these factors could ultimately affect the occurrence of sequelae. Additionally, children living in more distant areas may face greater barriers to accessing timely care.

A systematic review [16] emphasizes the importance of conducting more comprehensive studies to explore the effects of trauma in primary dentition on permanent teeth. Cohort studies with extensive sample sizes are particularly suited to deepen understanding of these outcomes. Prior cohort studies [11, 17] have documented significant findings on sequelae in permanent teeth, tracking them from the moment the trauma occurred in primary dentition. More recently, a classification system was introduced to categorize the severity of sequelae, further underscoring the necessity for additional research [18]. The primary aim of this study is to investigate the prevalence and severity of sequelae in anterior permanent teeth of children who suffered TDI in primary dentition and received care at a specialized dental trauma center. Furthermore, it seeks to identify factors related to the occurrence of developmental disturbances in permanent dentition.

## 2 Material and Methods

### 2.1 Study Settings and Design

This retrospective cohort study utilized clinical records and radiographs of children treated for dental injuries at the Traumatic Dental Injury Treatment Center for Primary Dentition (abbreviated as NETRAD in Portuguese), located at the Pediatric Dentistry Clinic of the School of Dentistry, Federal University of Pelotas, Brazil during the period from May 2002 to June 2016. This clinic provides access, treatment, and follow‐up assistance for TDIs in primary dentition, until the eruption of permanent teeth. The institutional Human Research Ethics Committee approved the study (Protocol 187/2011), and all participants' parents or legal guardians provided written informed consent. The study adhered to the Strengthening the Reporting of Observational Studies in Epidemiology (STROBE) guidelines [19].

The records of the patients were included in this study if provided detailed documentation of traumatic dental injuries (TDI) and at least two follow‐up visits registered. To be included, participants needed to have fully erupted permanent successor teeth with complete root formation at the time of data collection. Cases with incomplete dental records, low‐quality radiographic images, or the presence of caries in traumatized primary teeth were excluded from the analysis.

### 2.2 Data Collection

The patient care protocol at the dental trauma center follows the recommendations of the International Association of Dental Traumatology (IADT) Guidelines [20, 21]. Parents or legal guardians first participate in an interview to provide information about the child's medical and dental trauma history. A clinical examination is then performed, followed by a radiographic assessment if indicated by the type of TDI. Follow‐up appointments are scheduled based on the TDI classification.

A trained researcher gathered patient data, extracting details from clinical records, including age at the time of TDI, sex (male/female), residential area (Fragata, city center, Areal, Três Vendas, others or another city), location of the trauma (home, school, street, other places), delay in seeking treatment, number of affected teeth, and evidence of sequelae in permanent teeth.

The classification of sequelae in permanent teeth followed the system defined by Andreasen and Andreasen [20], complemented by criteria from Assunção et al. [4]. Clinic sequelae included white or yellow‐brown discoloration of enamel (1), enamel hypoplasia (2), crown dilaceration (3), and eruption disturbances (4). Radiographic sequelae comprised root dilaceration or angulation (1), root duplication (2), odontoma‐like malformation (3), partial or complete arrest of root formation (4), and sequestration of permanent tooth germ (5). The severity of sequelae was categorized as mild (white or yellow‐brown discoloration of enamel and enamel hypoplasia), moderate (crown dilaceration, eruption disturbances, root dilaceration or angulation, and root duplication), and severe (odontoma‐like malformation, partial or complete arrest of root formation, and sequestration of permanent tooth germ) [18].

TDIs were grouped into categories of hard tissue and supporting tissue trauma. Hard tissue trauma included enamel fracture, enamel and dentin fracture, enamel, dentin, and pulp fracture, crown‐root fracture, radicular fracture, and alveolar fracture; supporting tissue trauma encompassed concussion, subluxation, lateral luxation, extrusive luxation, intrusive luxation, and avulsion. A pediatric dentistry specialist conducted clinical and radiographic evaluations of TDIs, adhering to the IADT guidelines [20, 21] for primary dentition injuries.

### 2.3 Training and Calibration Procedure

The researcher underwent proper training and calibration to conduct clinical and radiographic assessments aimed at identifying sequelae in permanent teeth. Radiographic evaluations were performed using an X‐ray viewer alongside a magnifying glass, and patient photographs routinely taken during treatment were also analyzed. To ensure reliability, intra‐examiner variability was measured using the kappa coefficient, based on 70 photographs and periapical radiographs representing different conditions. These results were benchmarked against a gold standard, yielding an inter‐examiner weighted kappa of 0.80 and an intra‐examiner weighted kappa of 0.97.

### 2.4 Data Analysis

**FIGURE 1:**
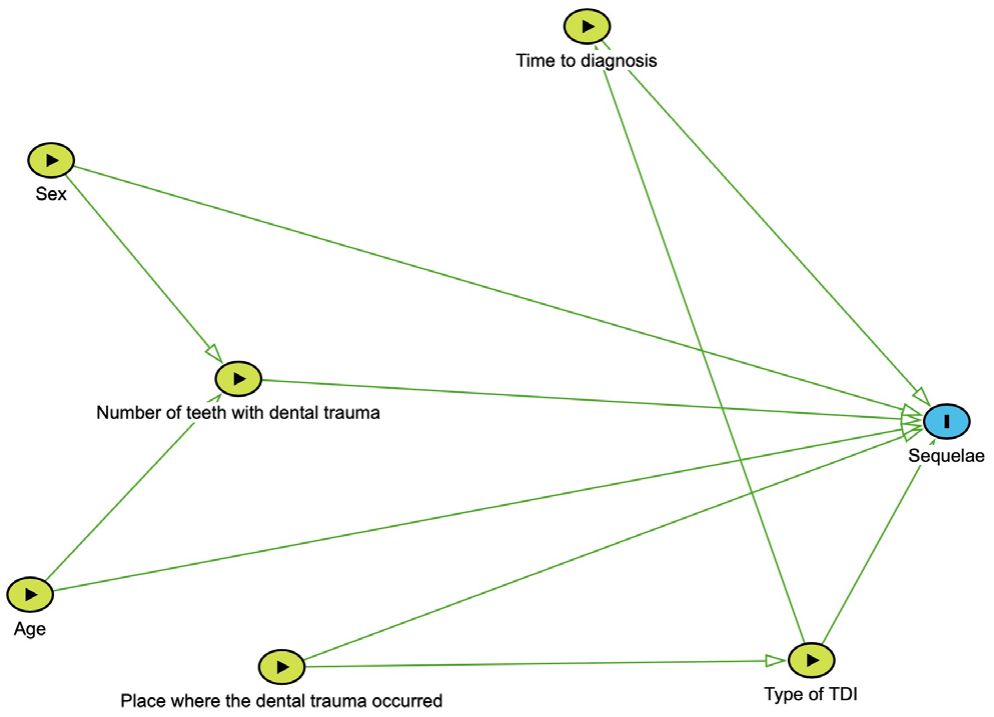
Directed Acyclic Graphs (DAG) showing the exposures and outcome (permanent teeth with sequelae). https://www.dagitty.net.

A directed acyclic graph (DAG) was used to select potential covariates for the analysis of the relationship between trauma in primary teeth and sequelae in permanent successors. The structured model included variables considered relevant for predicting sequelae. The DAG, created using the DAGitty software, incorporated confounding variables to be controlled in the final model, such as the child's age at the time of TDI, sex, number of affected teeth, place of occurrence, time elapsed between the injury and care seeking, and type of injury (Figure 1).

Data was analyzed using Stata version 14.0 (Stata Corp. LP, College Station, TX, USA). Descriptive statistics was performed to summarize data. Furthermore, relative risks (RR) and 95% confidence intervals (CI) for sequelae occurrence were calculated using Poisson regression with robust variance. A *p*‐value threshold of ≤ 0.05 was established to determine statistical significance.

## 3 Results

**FIGURE 2:**
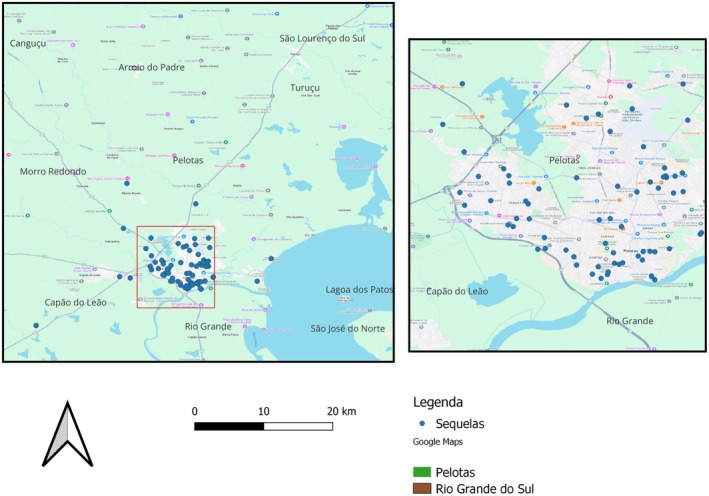
Distribution of sequelae cases in the city of Pelotas‐RS, Brazil.

A total of 931 dental records from treated patients were evaluated. Among them, 472 children had permanent successors erupted. However, 331 cases were excluded due to presenting < 2 follow‐ups, dental caries, or incomplete records. The sample consisted of 140 children, representing 242 traumatized primary teeth. Among these, 76 (54.3%) were boys and 64 (45.7%) were girls. The age distribution at the time of traumatic dental injury (TDI) was as follows: 28 (20%) were below 2 years, 60 (42.9%) were aged 2–4 years, and 52 (37.1%) were older than 4 years. In relation to the areas where the children lived, most were in Fragata (24.0%) and the city center (17.0%) (Figure 2). In terms of the number of affected teeth, 60 (42.9%) had one affected tooth, 60 (42.9%) had two affected teeth, and 20 (14.2%) had three to five affected teeth. Regarding the severity of permanent sequelae, out of the total cases, 66 (81.5%) were classified as mild, 13 (16.0%) as moderate, and 2 (2.5%) as severe (Table 1).

**TABLE 1:** Profile of patients with trauma to the primary dentition (*N* = 140).

	*N* (%)
Sex	
Male	76 (54.3)
Female	64 (45.7)
Age	
0–2 years	28 (20.0)
2–4 years	60 (42.9)
> 4 years	52 (37.1)
Area^a^	
City center	17 (17.0)
Areal	15 (15.0)
Três vendas	14 (14.0)
Fragata	24 (24.0)
Others	22 (22.0)
Another city	8 (8.0)
Number of teeth involved	
1	60 (42.9)
2	60 (42.9)
3 or more	20 (14.2)
Severity of permanent sequelae	
Mild	66 (81.5)
Moderate	13 (16.0)
Severe	2 (2.5)

^a^ Missing information for 40 children.

Among the 242 traumatized primary teeth, 81 (33.5%) of the permanent successors exhibited sequelae. The most prevalent type of sequela in the permanent teeth was discoloration of enamel. The types of dental trauma that most frequently caused sequelae in the permanent teeth were subluxation and intrusive luxation (Table 2). Additional details on the types of sequelae observed, according to the affected tooth and the child's age at the time of trauma, are provided in Table S1.

**TABLE 2:** Occurrence of sequelae in permanent teeth according to TDI suffered in primary teeth (*n* = 242).

	Presence of sequelae, *n* (%)	Absence of sequelae, *n* (%)	Type of sequelae					
			Discoloration of enamel, *n* (%)	Enamel hypoplasia, *n* (%)	Crown dilaceration, *n* (%)	Eruption disturbance, *n* (%)	Root dilaceration, *n* (%)	Odontoma‐like malformation, *n* (%)
Total	81 (33.47)	161 (66.53)	43 (17.77)	23 (9.50)	6 (2.48)	1 (0.41)	6 (2.48)	2 (0.83)
TDI								
Enamel fracture	3 (18.75)	13 (81.25)	3 (17.65)	—	—	—	—	—
Enamel and dentin fracture	2 (22.22)	7 (77.78)	—	1 (11.11)	—	—	1 (11.1)	—
Enamel, dentin and pulp fracture	1 (16.67)	5 (83.33)	1 (16.67)	—	—	—	—	—
Crown‐root fracture	3 (20.00)	12 (80.00)	1 (6.67)	—	—	—	1 (6.67)	1 (6.67)
Concussion	3 (21.43)	11 (78.57)	—	3 (21.43)	—	—	—	—
Subluxation	23 (37.10)	39 (69.60)	14 (22.95)	6 (9.84)	1 (1.64)	1 (1.64)	1 (1.64)	—
Lateral luxation	4 (14.81)	23 (85.19)	2 (7.41)	1 (3.70)	1 (3.70)	—	—	—
Intrusive luxation	24 (53.33)	21 (46.67)	13 (28.89)	6 (13.33)	3 (6.67)	—	1 (2.22)	1 (2.22)
Extrusive luxation	5 (33.33)	10 (66.67)	3 (20.0)	2 (13.33)	—	—	—	—
Avulsion	13 (39.39)	20 (60.61)	6 (18.18)	4 (12.12)	1 (3.03)	—	2 (6.06)	—

Table 3 presents the crude and adjusted Poisson regression analysis considering the presence of permanent teeth with sequelae as the outcome of the study. In the crude regression analysis, the child's age, the location where the trauma occurred, and the type of trauma remained associated with the outcome. After adjustment, considering the variables of the theoretical model proposed in the study, the same variables from the crude regression analysis remained associated with the outcome. Regarding the child's age, the frequency of sequelae in permanent teeth decreased as the age of the children evaluated in the study increased when compared to children under 2 years old [age 2–4 years: RR = 0.46; 95% CI (0.26–0.85) and over 4 years old—RR = 0.30; 95% CI (0.15–0.60); *p* = 0.003]. Regarding the location of the trauma, children who suffered dental trauma in the street or places other than their own home had a higher occurrence of sequelae in permanent teeth [RR = 2.07; 95% CI (1.16–3.66)]. Finally, considering the type of trauma in the primary dentition, teeth with trauma to the supporting tissue increased the probability of sequelae in the permanent teeth when compared to those teeth with trauma to hard tissue.

**TABLE 3:** Poisson regression analysis of the presence of sequelae in permanent teeth according to the variables of the proposed model in the study (*n* = 242 teeth).

Variable	Sequelae in permanent teeth					
	Crude			Adjusted		
	RR	95% CI	*p*	RR	95% CI	*p*
Sex						
Male	1.00		0.886	—	—	—
Female	0.97	0.68–1.39		—	—	
Age						
< 2 years	1.00		0.006	1.00		0.003
2–4 years	0.63	0.42–0.95		0.46	0.26–0.85	
> 4 years	0.49	0.31–0.77		0.30	0.15–0.60	
Number of teeth with dental trauma						
Up to 1 tooth	1.0		0.300	—		—
More than 1 tooth	1.27	0.81–1.99		—	—	
Time to diagnosis						
Up to 1 day	1.0		0.861	—		—
Up to 1 month	0.89	0.56–1.41		—	—	
More than 1 month	0.90	0.49–1.65		—	—	
Place where the dental trauma occurred						
Home	1.0		0.032	1.0		0.012
School	0.62	0.34–1.13		0.88	0.42–1.82	
Street/Other location	1.45	1.02–2.31		2.07	1.16–3.66	
Type of TDI						
Hard tissue	1.00		0.037	1.00		0.027
Supporting tissue	1.93	1.04–3.57		2.79	1.12–6.93	

## 4 Discussion

The present study assessed the impact of TDI in primary dentition on the permanent successors. The findings revealed that nearly 1/3 of the permanent teeth presented sequelae, indicating an important frequency and highlighting the importance of close monitoring of children with traumatized primary teeth. The age of the child at the time of trauma, the type of TDI, and the place where the dental trauma occurred were associated with developmental disturbances, emphasizing the importance of protecting primary teeth to ensure the integrity of the permanent dentition.

The risk for sequelae decreased with child age, in accordance with previous studies [3, 6, 22, 23, 24]. Younger children at the time of TDI face a greater risk of sequelae impacting permanent successors. This emphasizes the concern regarding trauma in early childhood [13] as it coincides with incomplete bone development and the early stages of odontogenesis in permanent teeth. Considering the stages of Nolla's development, younger children experiencing trauma during stages 1–3 of Nolla may have long‐term effects on the crown of the developing permanent tooth [2]. Nonetheless, crown sequelae may occur at any age, as enamel maturation and mineralization continue after initial root formation [3, 7]. The most significant damage to the root is observed in children aged 4–7 years, with root formation at stages 4–6 of Nolla [2].

The type of injury plays a significant role in the occurrence of sequelae in permanent dentition [2, 4, 25]. Findings from this study show that supporting tissue injuries were most commonly linked to sequelae in permanent teeth, aligning with earlier research [4, 7]. Damage to the permanent tooth germ may result from various mechanisms, including direct contact, follicular displacement, disruption of the enamel epithelium, or infections that increase neutrophil activity in surrounding bone, subsequently harming the enamel epithelium [5, 7]. Additionally, characteristics of children, like the high resilience of the alveolar bone and the larger trabecular spaces during early childhood, influence the likelihood of sequelae development [26, 27]. Finite element analysis from a previous study suggested that traumatic forces during TDI on primary teeth primarily affect the dental follicle and surrounding bone tissues, which can significantly impact the formation of permanent successors [28].

Frequency of sequelae was higher for teeth with avulsion or intrusive luxation. However, in the present study, nearly all types of injury caused sequelae in permanent successors, including concussion and subluxation, confirming a previous study [14]. The occurrence of sequelae following mild trauma may be a consequence of pulp necrosis and/or periapical inflammation in the primary tooth [14] or from the incorporation of hemoglobin breakdown products due to bleeding in the traumatized area [29]. These findings support that even minor TDIs are of considerable importance, as they may lead to unexpected consequences [30]. Lenzi et al. [14] found the highest prevalence of sequelae, specifically enamel hypoplasia, following intrusion. This observation can be attributed to the nature of intrusion, which pushes the primary teeth toward the germ of the permanent teeth, potentially causing severe sequelae, particularly during the early stages of permanent tooth development. Avulsion, which involves complete displacement of the tooth out of its alveolar socket, also may disrupt the normal developmental processes of the underlying permanent tooth germ. Del Negro et al. [13] evaluated the impact of avulsion of primary incisors and found a similar frequency of sequelae (44.5%) highlighted that the movement of the tooth during avulsion, combined with the close proximity between the primary tooth and the germ of the developing successor, can disrupt its subsequent growth and maturation.

The severity of sequelae was assessed, with mild defects being the most prevalent and severe sequelae the least frequent, consistent with findings from previous studies [18, 31]. The most prevalent clinical sequel observed in this study was discoloration of enamel, also in line with previous research [2]. Enamel hypoplasia was the second most common clinical sequelae, further emphasizing its high prevalence [11, 12, 32]. The predominance of these enamel alterations over other sequelae can be attributed to the fact that enamel discoloration and hypoplasia can result from less severe trauma in the deciduous dentition [33]. The wide age range of trauma in primary dentition causing enamel discolorations in permanent teeth is also related to the prolonged enamel maturation until eruption and hence can be damaged by blood derivatives [29].

When analyzing the distribution of children with sequelae, it was observed that most resided in the Fragata and city center neighborhoods, which are closer to the university, confirming that it is possible that children from more distant areas face greater difficulties in accessing care [13, 15]. Implementing prevention, control, and treatment strategies in areas with high dental trauma rates could enhance healthcare outcomes and efficiency, thus providing healthcare refinement [15]. In the present study, injuries occurring outside the home had a higher frequency of sequelae, also emphasizing the relevance of the environmental context in accidents. Aminu et al. [34] also highlight how educational interventions in schools can reduce the prevalence of dental trauma, especially in environments where children are more exposed to risks. The need for an integrated approach to improve access to dental care in vulnerable areas has also been addressed in studies [15, 34] exploring the relationship between geographic location and oral health conditions.

One of the strengths of this study is the long‐term, standardized follow‐up protocols employed in the dental trauma service, which allowed for the early detection and management of sequelae. Nevertheless, the study is not without limitations. As a retrospective cohort study relying on clinical records, a certain level of information bias is unavoidable, as some patients were excluded due to missing or low‐quality data. Additionally, files were not specifically designed for the study. To establish higher levels of scientific evidence regarding the effects of trauma in primary dentition on permanent teeth, more high‐quality, prospective, controlled studies are warranted [16].

This study contributes valuable insights into the long‐term consequences of TDI in primary dentition and the subsequent effects on permanent teeth, emphasizing the necessity for vigilant clinical and radiographic monitoring to address this significant issue effectively. In conclusion, an important frequency of sequelae in permanent teeth following trauma in primary dentition was found, although most were of mild severity. Trauma to supporting tissues, younger age (< 2 years), and injuries occurring outside the home were associated with the development of sequelae in permanent teeth.

## Author Contributions

All authors contributed to data collection and manuscript writing. Marilia Leão Goettems, Alexandre Emidio Ribeiro Silva, and Vanessa Polina Pereira da Costa were also responsible for data analysis. All authors reviewed and approved the final version of the manuscript.

## Ethics Statement

The study was approved by the local Human Research Ethics Committee of the Federal University of Pelotas (Protocol 187/2011) and all the participants signed informed consent.

## Conflicts of Interest

The authors declare no conflicts of interest.

## Supporting information


**Table S1.** Type of sequelae observed according to affected tooth and age at the time of trauma.

## Data Availability

The data that support the findings of this study are available from the corresponding author upon reasonable request.
