# Supplementary material for: Impact of Traumatic Injuries to Primary Teeth on the Development of Permanent Dentition: Findings From a Retrospective Cohort
Source: Dent Traumatol. 2025 Jul 25;42(1):63–70. doi: 10.1111/edt.70004 (PMC12803680; doi:10.1111/edt.70004)
Supplement: Supplementary file 1 — Table S1. Type of sequelae observed according to affected tooth and age at the time of trauma. [file EDT-42-63-s001.docx]

**Supplementary material**

Supplementary Table 1. Type of sequelae observed according to affected tooth and age at the time of trauma

|  | Type of sequelae | | | | | |
| --- | --- | --- | --- | --- | --- | --- |
|  | Discoloration of enamel  n (%) | Enamel hypoplasia  n (%) | Crown Dilaceration  n (%) | Eruption disturbance  n (%) | Root dilaceration  n (%) | Odontoma-like malformation  n (%) |
| Total | 43 (53.09) | 23 (28.40) | 6 (7.41) | 1 (1.23) | 6 (7.41) | 2 (2.47) |
| **Traumatized tooth** |  |  |  |  |  |  |
| Central upper incisors | 37 (56.06) | 15 (22.73) | 5 (7.58) | 1 (1.52) | 6 (9.09) | 2 (3.03) |
| Lateral upper incisors | 3 (30.00) | 6 (60.00) | 1 (10.00) | - | - | - |
| Canine upper | - | 1 (100.00) | - | - | - | - |
| Central lower incisors | 2 (66.67) | 1 (33.33) | - | - | - | - |
| Lateral lower incisors | 1 (100.00( | - | - | - | - | - |
| **Age** |  |  |  |  |  |  |
| 0-2 years | 8 (42.11) | 8 (42.11) | 2 (10.53) | - | 1 (5.26) | - |
| 2-4 years | 24 (63.16) | 9 (23.68) | 3 (7.89) | - |  | 2 (5.26) |
| >4 years | 11 (45.83) | 6 (25.00) | 1 (4.17) | 1 (4.17) | 5 (20.83) |  |
